# Reductive Stress and Ferroptosis: Linking Insulin Signaling to Metabolic Dysfunction

**DOI:** 10.3390/biom16060848

**Published:** 2026-06-10

**Authors:** Udayakumar Karunakaran, Suma Elumalai

**Affiliations:** 1 Institute of Medical Science, Yeungnam University College of Medicine, Daegu 42415, Republic of Korea; 2 Department of Genetics, Heersink School of Medicine, UAB Comprehensive Diabetes Center, University of Alabama at Birmingham, Birmingham, AL 35294, USA

**Keywords:** reductive stress, oxidative stress, insulin resistance, ferroptosis, lipid peroxidation, glutathione peroxidase 4 (GPX4), mitochondrial dysfunction, pentose phosphate pathway, metabolic disease

## Abstract

Reductive stress, characterized by excessive reducing equivalents such as NADH, NADPH, and reduced glutathione (GSH), is increasingly recognized as a pathophysiological counterpart to oxidative stress. Chronic hyperinsulinemia and insulin resistance promote this over-reduced state by increasing glucose flux, pentose phosphate pathway activity and de novo lipogenesis, thereby elevating NADPH pools and reshaping cellular lipid composition. While reducing equivalents are essential for biosynthesis and antioxidant defense, persistent over-reduction disrupts redox balance, mitochondrial function and metabolic flexibility. Paradoxically, this reductive metabolic environment may increase susceptibility to ferroptosis, an iron-dependent form of regulated cell death driven by lipid peroxidation and failure of glutathione peroxidase 4 (GPX4). Here, we define how reductive stress becomes deregulated in the context of insulin signaling and insulin resistance, and assess whether antioxidant interventions can mitigate ferroptosis, providing a framework for therapeutic strategies to restore redox balance in metabolic disease.

## 1. Introduction

Reductive stress are not merely passive counterparts to oxidation but constitute an active, finely tuned dimension of cellular physiology. Under homeostatic conditions, reducing equivalents such as NADH, NADPH, and reduced glutathione (GSH) function as dynamic regulators of antioxidant defense, biosynthesis, and redox signaling rather than simple electron donors [1]. NADPH, primarily generated via the pentose phosphate pathway, acts as a central metabolic currency that fuels both the glutathione and thioredoxin systems, thereby enabling precise detoxification of reactive oxygen species (ROS) while preserving redox-sensitive signaling cascades [2]. Importantly, transient excursions toward a more reduced intracellular environment are not inherently pathological; instead, they represent adaptive responses to states such as hypoxia, nutrient excess, or rapid proliferation, where enhanced reductive capacity supports macromolecular synthesis, repair, and metabolic plasticity [3]. In this light, reductive tone emerges as a dynamic and context-dependent variable, continuously counterbalanced by NAD^+^ regeneration, mitochondrial respiration, and controlled oxidant production to sustain redox equilibrium. However, this adaptive reductive stress can transition into pathology when regulatory thresholds are exceeded. Pathological reductive stress arises not simply from increased reducing equivalents but from their persistent accumulation beyond the cell’s buffering and regulatory capacity, resulting in chronically elevated NADH/NAD^+^ and NADPH/NADP^+^ ratios that can impair mitochondrial electron transport chain efficiency, increase electron leakage, and promote secondary ROS generation [4,5,6]. In contrast to the classical paradigm that links disease primarily to oxidative stress, this perspective highlights that excessive reduction can be equally disruptive. Sustained over-reduction interferes with disulfide bond formation, destabilizes proteostasis, and drives over-reduction of mitochondrial electron transport chain components, paradoxically promoting electron leak and secondary ROS generation [7]. Thus, reductive stress reveals a counterintuitive principle: an overabundance of antioxidant potential can itself become a source of oxidative damage. Chronic activation of anabolic and reducing pathways—such as those associated with hyperglycemia, hyperinsulinemia, or genetic upregulation of antioxidant systems—further entrenches this maladaptive state [8]. Emerging evidence from experimental models links reductive stress to cardiomyopathy, metabolic disorders, and neurodegeneration, reframing redox imbalance as a bidirectional spectrum rather than a unidirectional shift toward oxidation [9]. Consequently, sustained reductive stress should be understood as a distinct and underappreciated mechanism of redox-mediated pathology. However, many of the underlying mechanistic relationships remain incompletely understood, and direct causal evidence in humans is still limited. Consequently, reductive stress should currently be regarded as an emerging conceptual framework that complements, rather than replaces, traditional oxidative stress paradigms.

At the systems level, redox homeostasis is best conceptualized as an integrated network in which electron flux, metabolic state, and signaling pathways converge. Central to this network are the pyridine nucleotide couples NAD^+^/NADH and NADP^+^/NADPH, which function as metabolic rheostats that coordinate oxidative and reductive processes. The NAD^+^/NADH pair predominantly supports catabolic metabolism by channeling electrons from nutrient oxidation into mitochondrial respiration for ATP production, whereas NADP^+^/NADPH is dedicated to reductive biosynthesis and antioxidant defense [10,11]. Through this division of labor, cells maintain a functional separation between energy production and redox buffering while allowing crosstalk between the two systems. This observation is consistent with the central premise of this review that metabolic pathways involved in biosynthesis, antioxidant defense, and mitochondrial function are tightly integrated within a dynamic redox network. Within this framework, the glutathione system represents the principal thiol-based buffering axis of the cell. Comprising GSH, glutathione reductase (GR), and glutaredoxins, it operates as a highly responsive redox reservoir. GSH directly neutralizes hydrogen peroxide and lipid peroxides via glutathione peroxidases, generating oxidized glutathione (GSSG), which is efficiently recycled back to GSH by GR in an NADPH-dependent manner [11,12]. The GSH/GSSG ratio thus serves not only as a marker of redox status but also as a regulatory node influencing signaling pathways and cellular susceptibility to damage. Notably, mitochondrial glutathione (mGSH) provides compartment-specific protection, safeguarding electron transport chain integrity and modulating cell death pathways such as apoptosis and ferroptosis under conditions of redox imbalance.

**Figure 1:**
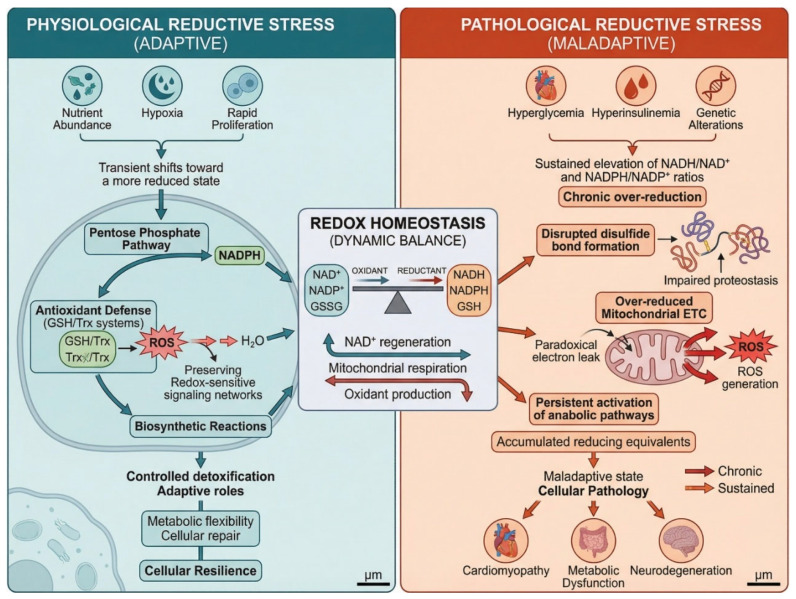
Reductive stress in physiological versus pathological contexts. (**Left**) Adaptive reductive stress arises from nutrient abundance, hypoxia, or proliferation, causing transient increases in reducing equivalents that support antioxidant defense, biosynthesis, and redox signaling. (**Center**) Redox homeostasis reflects a dynamic balance between oxidants and reductants, maintained by mitochondrial respiration and NAD^+^ regeneration. (**Right**) Pathological reductive stress, driven by hyperglycemia, hyperinsulinemia, and genetic alterations, leads to sustained over-reduction, disrupting proteostasis and mitochondrial function, increasing ROS, and promoting cardio-metabolic and neurodegenerative disease.

Complementing this system, the thioredoxin (Trx) network introduces an additional layer of regulatory precision. Composed of Trx, thioredoxin reductase (TrxR), and NADPH, it functions as a versatile thiol oxidoreductase system that maintains proteins in their reduced states and directly supports peroxiredoxins in hydrogen peroxide detoxification [13,14]. Beyond antioxidant defense, reduced Trx plays essential roles in DNA synthesis and repair, protein folding, and the modulation of redox-sensitive transcription factors, underscoring its integrative role in cellular function. The catalytic efficiency of TrxR, conferred by its selenocysteine residue, enables rapid recycling of oxidized Trx in both cytosolic and mitochondrial compartments. Rather than operating in isolation, the glutathione and thioredoxin systems are functionally interconnected, forming a resilient and partially redundant network. Crosstalk between these pathways allows compensation under conditions of stress, enhancing cellular robustness against redox perturbations [15,16]. At the core of this integration lies NADPH, which serves as the unifying electron donor that couples metabolic activity to antioxidant capacity. Perturbations in NAD^+^/NADH or NADP^+^/NADPH ratios—arising from aging, metabolic dysfunction, or mitochondrial impairment—can therefore propagate through both systems, compromising their ability to maintain reduced states and leading to widespread disruptions in signaling and cellular integrity [17,18]. Collectively, these insights reposition redox homeostasis as a dynamic, multidimensional network rather than a simple balance between oxidants and antioxidants. Within this paradigm, both oxidative and reductive extremes are potentially deleterious, and cellular resilience depends on the precise orchestration of nucleotide cofactors, thiol systems, and metabolic fluxes to sustain redox equilibrium in a fluctuating environment (Figure 1).

## 2. Insulin Signaling Promotes Reductive Stress

**Figure 2:**
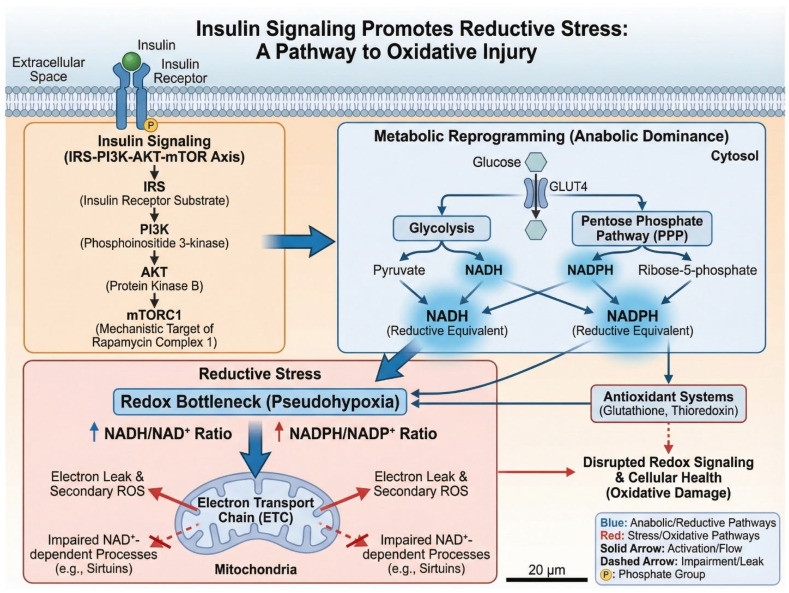
Insulin signaling promotes reductive stress leading to oxidative injury. Insulin activates the IRS-PI3K-AKT-mTORC1 pathway, driving metabolic reprogramming toward increased glycolysis and pentose phosphate pathway flux, elevating NADH and NADPH production. This anabolic dominance creates a redox bottleneck characterized by increased NADH/NAD^+^ and NADPH/NADP^+^ ratios. The resulting over-reduced state impairs mitochondrial electron transport chain function, promotes electron leak and ROS generation, and disrupts NAD^+^-dependent processes. Concurrently, antioxidant systems (glutathione and thioredoxin) become dysregulated, leading to disrupted redox signaling and oxidative damage.

Insulin signaling can be reinterpreted not merely as a classical regulator of glucose homeostasis, but as a central systems-level architect of the cellular redox landscape, integrating nutrient sensing with electron flow control and mitochondrial metabolic capacity. Through activation of the IRS-PI3K-AKT-mTOR axis, insulin promotes a coordinated anabolic program that redirects carbon flux toward glycolysis, the pentose phosphate pathway (PPP), and lipid biosynthesis [19,20]. In this framework, insulin functions as a “redox programming node,” dynamically regulating intracellular electron distribution according to nutrient availability and growth signals. This anabolic reprogramming increases the production and compartmentalization of reducing equivalents, particularly NADH and NADPH, thereby shifting cells toward reductive stress rather than simply enhancing antioxidant defense. Elevated NADH/NAD^+^ ratios create a functional redox disequilibrium in which electron supply exceeds mitochondrial oxidative phosphorylation capacity, generating pseudo-hypoxic signaling despite normoxic conditions and limiting NAD^+^-dependent processes such as sirtuin activity, DNA repair, and mitochondrial biogenesis [21]. Simultaneously, insulin-driven PPP activation elevates NADPH availability, supporting biosynthetic pathways and antioxidant systems, including glutathione and thioredoxin networks [22]. While adaptive during acute nutrient abundance, persistent hyperinsulinemia may drive a chronic hyper-reduced state that diminishes redox signaling flexibility and disrupts cellular homeostasis. This perspective challenges the classical oxidative stress paradigm by positioning reductive stress as an upstream driver of oxidative injury. Excess NADH increases electron pressure on the mitochondrial electron transport chain, enhancing electron leakage and secondary ROS formation, whereas sustained NADPH excess may maintain antioxidant systems in an over-reduced state that suppresses physiological ROS signaling required for adaptive stress responses [23,24]. Insulin further amplifies reductive stress by suppressing fatty acid oxidation and autophagy, thereby limiting NAD^+^ regeneration and reducing the cell’s capacity to dissipate excess reducing equivalents [20,21]. Over time, this establishes a self-reinforcing network in which anabolic signaling, mitochondrial dysfunction, and redox disequilibrium perpetuate metabolic inflexibility. The resulting intracellular environment—characterized by elevated NADH, NADPH, and lipid intermediates—predisposes cells to lipid peroxidation, ferroptotic susceptibility, and progressive mitochondrial impairment under metabolic stress [25,26]. Within insulin-resistant states, chronic nutrient excess combined with impaired mitochondrial oxidative capacity sustains accumulation of reducing equivalents and amplifies secondary ROS generation [5]. In this context, reductive stress may represent an early initiating event preceding overt oxidative damage, reshaping the temporal hierarchy of metabolic dysfunction. This framework emphasizes metabolic flexibility and efficient electron throughput regulation, rather than maximal antioxidant capacity alone, as central determinants of cellular homeostasis [23,24]. Future strategies may therefore focus on restoring redox oscillatory dynamics through NAD^+^ replenishment, enhancement of fatty acid oxidation and mitophagy, or precision modulation of mitochondrial electron flow. Collectively, this framework positions insulin as a master regulator of nutrient-responsive redox programming and suggests that cellular redox homeostasis depends on the precise alignment between electron supply and utilization across metabolic networks. Disruption of this balance—whether biased toward oxidation or reduction—leads to systemic instability. Recognizing insulin-driven reductive stress as a primary and potentially targetable event opens new conceptual and therapeutic frontiers, including redox-guided precision medicine, mitochondrial flux engineering, and interventions aimed at restoring electron homeostasis in chronic metabolic disease (Figure 2).

## 3. Insulin Resistance Promotes Reductive Stress

Insulin resistance and compensatory hyperinsulinemia represent core features of metabolic disorders such as type 2 diabetes mellitus (T2DM) and metabolic syndrome, which are deeply intertwined with cellular redox dysregulation and impaired antioxidant network resilience. At a fundamental level, insulin resistance is a state in which peripheral tissues—especially skeletal muscle, liver, and adipose tissue—fail to respond adequately to normal insulin signaling, necessitating increased insulin production from pancreatic β-cells to maintain glucose homeostasis [27]. From a systems biology and future medicine perspective, this compensatory endocrine state should be viewed not merely as a glycemic adaptation but as a persistent metabolic signal that progressively rewires intracellular redox circuits and electron flow architecture across multiple organ systems. Chronic hyperinsulinemia not only marks disease progression but also drives metabolic alterations that significantly perturb redox homeostasis, particularly transitions in the NAD^+^/NADH and NADP^+^/NADPH couples and antioxidant systems such as glutathione and thioredoxin. Moreover, in insulin-resistant states, chronic nutrient oversupply and impaired substrate oxidation lead to excessive NADH accumulation and a relative depletion of NAD^+^, thereby increasing the NADH/NAD^+^ ratio and functionally restricting NAD^+^-dependent metabolic and signaling pathways. This phenomenon manifests as pseudohypoxia, a redox-defined state in which intracellular electron saturation mimics hypoxic signaling despite adequate oxygen availability, thereby impairing mitochondrial efficiency, metabolic flexibility, and adaptive stress responses [27,28]. Elevated NADH levels further impair mitochondrial electron transport chain dynamics, particularly at complex I, enhancing electron leakage and promoting the formation of reactive oxygen species (ROS). In parallel, NAD^+^ depletion compromises the activity of NAD^+^-dependent enzymes such as sirtuins, which regulate mitochondrial biogenesis, fatty acid oxidation, and stress resistance pathways, thereby amplifying insulin resistance through epigenetic and post-translational modifications [28,29,30]. Moreover, cells attempt to counteract excessive NADH accumulation through compensatory pathways such as increased glycerol secretion, which promotes NADH oxidation but is energetically costly and leads to ATP depletion and reduced glucose efficiency. This energy deficit activates AMP-activated protein kinase (AMPK), which restores metabolic balance by enhancing ATP-generating pathways and inhibiting ATP-consuming processes, including feedback regulation of glycerol synthesis. In the context of insulin resistance, chronic nutrient overload and impaired mitochondrial oxidation sustain elevated NADH levels, making these compensatory responses persistent and energetically burdensome. This links reductive stress to progressive metabolic dysfunction, as redox imbalance both reflects and reinforces the energetic and signaling defects characteristic of insulin-resistant states [31].

In insulin resistance, mitochondrial dysfunction and excessive NADH-driven ROS production increase GSH utilization, while impaired NADPH allocation compromises its regeneration capacity, ultimately lowering the GSH/GSSG ratio. This redox disequilibrium contributes to oxidative damage in insulin-sensitive tissues and disrupts redox-sensitive post-translational modifications of insulin signaling components, including insulin receptor substrates and GLUT4 translocation machinery, thereby exacerbating glucose uptake impairment and reinforcing systemic insulin resistance [27]. Future antioxidant strategies are likely to evolve from simple supplementation toward dynamic redox cycling therapies aimed at restoring GSH turnover kinetics rather than static pool restoration. Indeed, the thioredoxin (Trx) system constitutes a second major redox regulatory axis, comprising thioredoxin, thioredoxin reductase (TrxR), and NADPH. This system regulates protein disulfide reduction and supports peroxiredoxin-mediated peroxide detoxification. A critical modulator of this system is thioredoxin-interacting protein (TXNIP), which is upregulated under hyperglycemic and oxidative stress conditions, leading to Trx inhibition and amplification of oxidative stress, inflammasome activation, and pancreatic β-cell dysfunction [32]. In insulin-resistant states, increased ROS burden and NADPH demand place the Trx system under sustained stress, diminishing its capacity to maintain redox signaling fidelity [32]. The interplay between these redox systems reveals a tightly coupled network in which disturbances in one compartment propagate system-wide metabolic dysfunction. Importantly, this dysregulation also intersects with reductive stress, as altered NADH/NAD^+^ and NADPH flux can simultaneously drive excessive reducing pressure while overwhelming antioxidant recycling capacity, leading to a paradoxical state of both reductive and oxidative imbalance. When these antioxidant networks fail to compensate, redox-sensitive signaling proteins involved in insulin action—such as kinases, phosphatases, and transcriptional regulators—become functionally impaired, further weakening insulin responsiveness and metabolic control [27]. Additionally, chronic hyperinsulinemia exacerbates this process by continuously driving substrate influx and anabolic signaling, thereby reinforcing redox imbalance and metabolic rigidity. Insulin resistance (IR) can promote ferroptosis by disrupting metabolic, redox, and iron homeostasis, thereby creating conditions that favor iron-dependent lipid peroxidation and cell death. In insulin-resistant states, chronic metabolic overload (hyperglycemia, elevated free fatty acids) increases mitochondrial and cytosolic ROS production while simultaneously impairing NADPH-generating pathways such as the pentose phosphate pathway (PPP). Since NADPH is essential for regenerating reduced glutathione (GSH) and maintaining thioredoxin activity, its depletion weakens the GPX4-GSH antioxidant axis that normally prevents lipid peroxide accumulation. As a result, phospholipid polyunsaturated fatty acids become vulnerable to iron-dependent peroxidation, a defining feature of ferroptosis [33].

In addition, IR is associated with impaired insulin signaling through PI3K–AKT pathways, which can indirectly affect glucose metabolism and reduce flux through NADPH-producing enzymes such as glucose-6-phosphate dehydrogenase (G6PD). Reduced NADPH availability also limits the activity of glutathione reductase and thioredoxin reductase, further compromising cellular redox buffering capacity. In this context, even moderate iron accumulation—commonly observed in metabolic syndrome and type 2 diabetes—can amplify Fenton chemistry and lipid radical formation. Therefore, NADPH deprivation acts as a metabolic bridge linking insulin resistance to increased susceptibility to ferroptosis by weakening antioxidant regeneration and enhancing lipid oxidative stress [34,35,36,37]. Importantly, this relationship is not purely linear; rather, IR creates a redox-impaired but pro-oxidant environment, where diminished NADPH-dependent defense systems coexist with elevated ROS generation. This imbalance shifts cells toward a state in which ferroptosis is more easily triggered, especially in metabolically active tissues such as liver, pancreas, and adipose tissue. Additionally, IR is linked to iron dysregulation through altered hepcidin–ferroportin signaling, which can increase intracellular iron availability and promote Fenton chemistry, amplifying oxidative damage. In metabolic tissues such as liver, adipose tissue, and pancreas, these combined effects make cells more vulnerable to ferroptotic injury [38].

**Figure 3:**
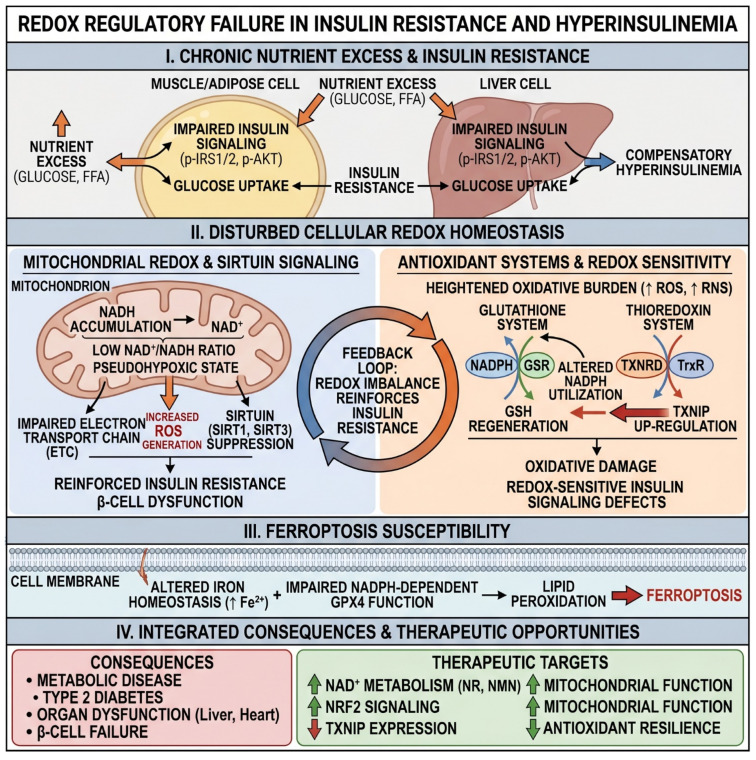
Redox regulatory failure in insulin resistance and compensatory hyperinsulinemia. Chronic nutrient excess promotes insulin resistance and compensatory hyperinsulinemia through impaired insulin signaling in metabolic tissues (**I**). Insulin-resistant states are associated with mitochondrial NADH accumulation, reduced NAD^+^ availability, impaired electron transport chain function, increased ROS production, and suppression of sirtuin signaling, leading to redox dysregulation and β-cell dysfunction (**II**). Simultaneously, disruption of the glutathione and thioredoxin antioxidant systems, driven by impaired NADPH-dependent recycling and TXNIP upregulation, weakens antioxidant defenses and reinforces insulin resistance through a self-perpetuating redox imbalance. These alterations increase susceptibility to ferroptosis via impaired GPX4 activity, disturbed iron homeostasis, and enhanced lipid peroxidation (**III**). Collectively, these mechanisms contribute to metabolic disease progression and identify potential therapeutic targets, including NAD^+^ restoration, NRF2 activation, TXNIP inhibition, and enhancement of antioxidant recycling pathways (**IV**).

From a translational perspective, growing evidence suggests that restoring redox equilibrium can significantly improve insulin sensitivity. NAD^+^ restoration strategies using precursors such as nicotinamide riboside or nicotinamide mononucleotide have demonstrated improvements in mitochondrial function and glucose homeostasis in experimental models [39]. Similarly, activation of NRF2-mediated transcriptional programs enhances endogenous antioxidant capacity, stabilizing redox networks and improving insulin signaling efficiency [40]. Targeted modulation of TXNIP and reinforcement of thioredoxin activity represent additional promising strategies, particularly for protecting pancreatic β-cells from glucolipotoxic stress and preserving endocrine function [32]. In summary, insulin resistance and compensatory hyperinsulinemia induce profound and interconnected disruptions in cellular redox homeostasis. Alterations in NADH/NAD^+^ and NADPH flux impair mitochondrial efficiency, elevate ROS production, and overwhelm glutathione and thioredoxin antioxidant systems. These redox disturbances, in turn, feed back onto insulin signaling pathways, exacerbating metabolic dysfunction and perpetuating disease progression. Collectively, this framework supports a future-oriented paradigm in which metabolic disease is understood not solely as a disorder of glucose regulation, but as a failure of electron flux coordination across cellular redox networks. Therapeutic strategies that restore redox couple balance, enhance mitochondrial flexibility, and reinforce antioxidant cycling capacity represent a promising frontier for preventing and treating insulin resistance and its associated metabolic complications (Figure 3).

## 4. Mechanistic Convergence of Insulin Signaling, Insulin Resistance, and Ferroptosis

The interplay between insulin signaling, insulin resistance, and ferroptosis can be reinterpreted as a tightly integrated, multi-layered control system in which metabolic flux, redox balance, and lipid architecture converge to determine cellular fate. Rather than functioning as isolated pathways, these processes form a dynamic and adaptive network governed by insulin as a central regulatory node. Through activation of the IRS-PI3K-AKT-mTOR axis, insulin orchestrates anabolic metabolism by enhancing glucose uptake, stimulating the pentose phosphate pathway, increasing NADPH production, and promoting lipid and protein biosynthesis [41,42,43]. In a forward-looking conceptual framework, insulin can thus be viewed as a metabolic–redox integrator that programs cellular electron flow, biosynthetic capacity, and membrane composition in response to nutrient availability. Under physiological conditions, this coordinated network supports growth, repair, and antioxidant defense. However, chronic or excessive insulin signaling—particularly in the context of nutrient excess—drives a persistent shift toward reductive stress. Elevated NADH/NAD^+^ ratios create a pseudohypoxic intracellular state that limits NAD^+^-dependent processes such as sirtuin-mediated mitochondrial biogenesis and fatty acid oxidation, thereby constraining metabolic flexibility and adaptive capacity [41,44]. From a future research perspective, this pseudohypoxic state may represent an early, quantifiable biomarker of metabolic inflexibility, detectable through advanced redox imaging and compartment-specific metabolite profiling. Simultaneously, sustained activation of the PPP elevates NADPH levels, maintaining glutathione and thioredoxin systems in a hyper-reduced state. While initially protective, this persistent reductive stress disrupts redox signaling fidelity and creates conditions in which reductive stress and reactive oxygen species (ROS) generation coexist [45]. This paradox highlights a key emerging principle: redox imbalance is not unidirectional but instead reflects a breakdown in the dynamic equilibrium between electron supply and utilization. Future therapeutic strategies may therefore focus on redox recalibration, aiming to restore oscillatory signaling rather than simply suppress oxidative stress. In insulin-resistant states, compensatory hyperinsulinemia further amplifies this imbalance. Impaired insulin receptor signaling reduces effective glucose utilization, yet sustained anabolic signaling continues to drive NADH and NADPH production, reinforcing reductive overload [45,46]. Elevated NADH increases electron pressure within the mitochondrial electron transport chain, promoting electron leakage and excessive ROS generation [47,48]. At the same time, NADPH is increasingly diverted toward lipogenesis, limiting its availability for antioxidant recycling. This dual burden compromises both the glutathione (GSH/GSSG) and thioredoxin (Trx/TrxR) systems. GSH depletion weakens detoxification of hydrogen peroxide and lipid peroxides, while upregulation of TXNIP inhibits thioredoxin activity, impairing protein thiol regulation and peroxiredoxin function [49,50,51]. Collectively, these disruptions establish a self-reinforcing feedback loop in which reductive stress and oxidative damage amplify one another, creating a fragile and highly reactive intracellular environment.

A critical downstream consequence of this convergence is increased susceptibility to ferroptosis, an iron-dependent, and lipid peroxidation-driven form of regulated cell death. Insulin-driven lipogenesis expands cellular pools of polyunsaturated fatty acids (PUFAs), which serve as substrates for lipid peroxidation. Concurrently, imbalances in NADPH and GSH compromise the activity of glutathione peroxidase 4 (GPX4), the key enzyme responsible for detoxifying lipid hydroperoxides [50,51]. Mitochondrial ROS generated under conditions of NADH excess further accelerate lipid peroxidation, while impaired glutathione and thioredoxin systems fail to neutralize accumulating oxidative damage [52]. From a systems perspective, ferroptosis emerges not as an isolated endpoint but as the integrated outcome of dysregulated metabolic flux, redox disequilibrium, and lipid remodeling. This mechanistic convergence is particularly significant in metabolically active and insulin-responsive tissues such as pancreatic β-cells, liver, and skeletal muscle, where high metabolic throughput and limited antioxidant reserves increase vulnerability. It underscores the concept that insulin signaling, insulin resistance, and ferroptosis are components of a unified regulatory network in which electron flow, redox buffering, and membrane composition are tightly coupled determinants of cellular survival [53,54,55]. Importantly, this framework reveals the nonlinear nature of redox biology: both excessive reduction (reductive stress) and uncontrolled oxidation (ROS accumulation) contribute to ferroptotic sensitivity, suggesting that oxidative damage in metabolic disease may originate upstream from reductive imbalance rather than from simple antioxidant deficiency. A direct mechanistic link between reductive stress and ferroptosis has not yet been fully established; however, emerging evidence suggests that reductive stress may modulate susceptibility to ferroptotic cell death through disruption of redox homeostasis rather than through direct induction. Reductive stress, defined by excessive accumulation of reducing equivalents such as NADPH, NADH, and reduced glutathione (GSH), can paradoxically impair cellular redox flexibility and adaptive antioxidant signaling. Although these reducing systems are essential for preventing lipid peroxidation, chronic reductive imbalance may desensitize redox-responsive pathways such as Nrf2 and disturb the dynamic cycling of NADPH-dependent antioxidant systems. Since ferroptosis is driven by iron-dependent accumulation of lipid hydroperoxides, its execution is tightly regulated by the glutathione–GPX4 axis, which requires balanced NADPH availability for GSH regeneration. In conditions of reductive stress, altered metabolic flux and mitochondrial dysfunction may still promote secondary reactive oxygen species generation, particularly when electron transport becomes overloaded or uncoupled, thereby creating localized oxidative bursts capable of initiating lipid peroxidation. Moreover, reductive stress may influence lipid metabolism by promoting incorporation of polyunsaturated fatty acids into membrane phospholipids, increasing the availability of substrates for iron-catalyzed peroxidation. Together, these alterations suggest that reductive stress does not directly trigger ferroptosis but instead creates a metabolically unstable state in which redox imbalance, impaired antioxidant responsiveness, and lipid remodeling collectively lower the threshold for ferroptotic cell death upon oxidative or iron-dependent insults (Table 1).

**Figure 4:**
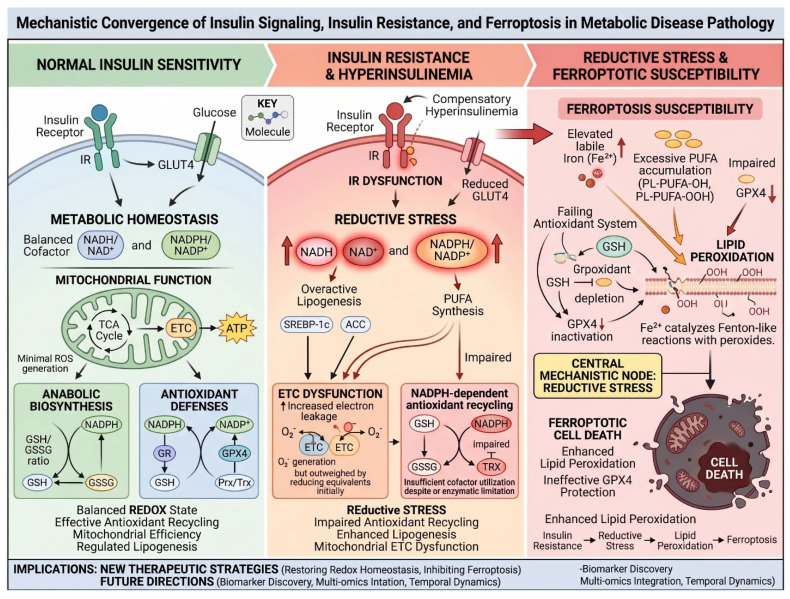
Mechanistic convergence of insulin signaling, redox balance, and ferroptosis. (**Left**) (normal): Intact insulin signaling (IRS-PI3K-AKT-mTOR) supports anabolic metabolism and glucose uptake, maintains low NADH/NAD^+^ ratio, efficient mitochondrial function, and minimal ROS. NADPH from the PPP sustains antioxidant defenses (glutathione and thioredoxin systems), preserving lipid homeostasis and preventing lipid peroxidation, thereby protecting against ferroptosis. (**Right**) (pathological): Impaired insulin signaling with compensatory hyperinsulinemia drives persistent anabolic flux, increasing NADH/NAD^+^ ratio (pseudohypoxia) and mitochondrial ROS. NADPH accumulation and TXNIP upregulation cause reductive stress, GSH depletion, and thioredoxin inhibition. Enhanced lipogenesis and PUFA accumulation provide substrates for lipid peroxidation. Together, ROS elevation and weakened antioxidant defenses promote lipid peroxide buildup and ferroptotic cell death.

Strengthening endogenous antioxidant systems, including glutathione and thioredoxin networks, improves cellular resilience to both reductive and oxidative stress, thereby restoring redox homeostasis and metabolic adaptability. Targeting these convergent nodes within an integrated metabolic–redox network offers a unified therapeutic strategy capable of simultaneously addressing insulin resistance, reductive stress, and ferroptosis susceptibility in metabolic disease. Overall, this framework reframes metabolic dysfunction as a systems-level failure in which insulin signaling dysregulation, redox imbalance, and lipid peroxidation are tightly interconnected. This perspective shifts the traditional emphasis away from oxidative stress alone and positions reductive stress as a critical upstream driver of metabolic injury. A clearer mechanistic understanding of this relationship will be critical for developing precision interventions aimed at restoring redox homeostasis and preventing downstream cellular injury (Figure 4).

## 5. Conclusions and Future Directions

The mechanistic convergence of insulin signaling, insulin resistance, and ferroptosis highlights a complex interplay between metabolic flux, redox homeostasis, and lipid peroxidation that underpins metabolic disease pathology. Normal insulin sensitivity maintains a delicate balance between NADH/NAD^+^ and NADPH/NADP^+^ ratios, supporting mitochondrial function, anabolic biosynthesis, and antioxidant defenses via glutathione and thioredoxin systems. In contrast, insulin resistance and compensatory hyperinsulinemia shift this balance toward persistent reductive stress, characterized by NADH/NAD^+^ and NADPH/NADP^+^ accumulation, impaired antioxidant recycling, and mitochondrial electron transport dysfunction. This dysregulated redox state, coupled with enhanced lipogenesis and polyunsaturated fatty acid accumulation, primes cells for lipid peroxidation and ferroptotic susceptibility. Thus, reductive stress emerges as a central upstream mediator linking insulin dysregulation to oxidative injury and regulated cell death, reframing the traditional paradigm that attributes metabolic pathology solely to oxidative stress. The integration of metabolic, redox, and lipid regulatory pathways underscores that therapeutic strategies must target multiple nodes to restore homeostasis effectively. Future directions should focus on delineating precise molecular nodes that coordinate NAD^+^/NADH and NADPH/NADP^+^ homeostasis under insulin-resistant conditions and identifying biomarkers that reflect early reductive stress prior to overt oxidative damage. Therapeutic exploration may include interventions to restore NAD^+^ balance, modulate NADPH availability, enhance glutathione and thioredoxin function, or inhibit ferroptosis through GPX4 activators or lipid-peroxidation scavengers. Additionally, multi-omics approaches integrating metabolomics, lipidomics, and redox proteomics could elucidate tissue-specific vulnerability to ferroptosis in metabolic disorders. Finally, understanding the temporal dynamics of insulin signaling, redox flux, and ferroptotic priming will be essential for developing targeted strategies that prevent metabolic complications while preserving adaptive redox responses. Overall, recognizing and therapeutically targeting the convergence of insulin signaling, reductive stress, and ferroptosis provides a compelling framework for next-generation interventions in metabolic disease. Modulation of this interconnected network offers opportunities to restore redox and bioenergetic balance, improve insulin sensitivity, and reduce lipid peroxidation-driven cell injury. Such convergence-based strategies may ultimately redefine how diabetes and its end-organ complications are conceptualized and treated, moving the field toward mechanism-guided, network-targeted therapeutics capable of altering disease trajectory rather than merely mitigating its downstream consequences.

## Figures and Tables

**Table 1:** Mechanistic convergence of insulin sensitivity, insulin resistance, and ferroptosis.

Mechanism	Insulin Sensitivity (Normal)	Insulin Resistance (Pathological)	Impact on Ferroptosis	References
Insulin Signaling	Activation of IRS-PI3K-AKT-mTOR pathway promotes glucose uptake, protein synthesis, and metabolic flexibility	Impaired IRS-PI3K-AKT signaling with compensatory hyperinsulinemia and persistent anabolic drive	Increased lipogenesis and membrane PUFA availability enhance susceptibility to lipid peroxidation	[19,27,40]
NADH/NAD^+^ Balance	Efficient NADH oxidation through mitochondrial respiration; NAD^+^ regeneration supports glycolysis, TCA cycle, β-oxidation, and sirtuin activity	Elevated NADH/NAD^+^ ratio induces reductive stress -pseudohypoxia, inhibits NAD^+^-dependent pathways, and promotes mitochondrial dysfunction	Increased mitochondrial electron pressure and ROS generation contribute to lipid peroxidation and ferroptotic susceptibility	[3,5,6,11,17,18,21,23,46]
NADPH/NADP^+^ Balance	NADPH generated through PPP and related pathways supports glutathione, thioredoxin, and antioxidant defenses	Altered NADPH flux during chronic hyperinsulinemia contributes to reductive stress and enhanced lipid synthesis; antioxidant demand may exceed capacity	Imbalance between ROS production and NADPH-supported detoxification promotes ferroptotic signaling	[22,23,24,25,29,30,31,51]
Glutathione System (GSH/GSSG)	Efficient recycling of GSSG to GSH maintains redox homeostasis and detoxifies lipid peroxides through GPX4	Chronic ROS production, impaired cysteine availability, and metabolic dysfunction reduce effective GSH-dependent protection	Reduced GPX4 activity permits phospholipid hydroperoxide accumulation, a hallmark of ferroptosis	[10,12,16,34,35,36,38,53]
Thioredoxin System (Trx/TrxR)	Maintains protein thiol homeostasis and supports peroxide detoxification through peroxiredoxins	TXNIP upregulation and impaired Trx activity weaken antioxidant defenses and amplify oxidative stress	Increased sensitivity to ferroptotic stimuli and propagation of oxidative damage	[13,14,15,33,49]
Lipid Metabolism	Balanced lipid synthesis and oxidation; controlled incorporation of PUFAs into membrane phospholipids	Hyperinsulinemia and insulin resistance increase lipogenesis and accumulation of oxidizable PUFA-containing phospholipids	ACSL4-dependent PUFA enrichment provides substrates for lipid peroxidation and ferroptosis	[27,30,41,52]
Mitochondrial Function	Efficient electron transport with limited ROS generation and effective ATP production	NADH excess, impaired electron transport, and metabolic overload increase ROS production and mitochondrial stress	ROS-driven lipid peroxidation amplifies ferroptotic vulnerability	[7,20,21,28,45]
Iron Homeostasis	Tight regulation of iron uptake, storage, and utilization minimizes oxidative injury	Dysregulated iron handling and increased labile iron pool may accompany metabolic dysfunction	Iron-catalyzed Fenton reactions drive lipid peroxide formation and ferroptosis execution	[34,35,36,41,42,47,53]
GPX4-FSP1 Ferroptosis Defense Axis	GPX4, glutathione, CoQ10/FSP1, and related systems suppress phospholipid peroxide accumulation	Antioxidant defenses become insufficient relative to oxidative burden and lipid peroxide generation	Failure of GPX4/FSP1 pathways directly triggers ferroptotic cell death	[35,44,50]
NRF2 Antioxidant Response	NRF2 regulates antioxidant genes, glutathione synthesis, and cellular stress adaptation	NRF2 responses may become insufficient or dysregulated during chronic metabolic stress	Reduced antioxidant resilience enhances ferroptotic susceptibility	[43,54]
Overall Redox Status	Balanced oxidative and reductive signaling enables metabolic adaptation and cellular resilience	Persistent reductive stress combined with localized oxidative bursts causes redox disequilibrium	Redox imbalance integrates metabolic dysfunction, lipid peroxidation, and ferroptotic signaling	[1,2,8,23,24,26,37,43]
Outcome	Metabolic homeostasis, insulin responsiveness, and protection against oxidative damage	Progressive insulin resistance, mitochondrial dysfunction, inflammation, and metabolic disease	Increased ferroptosis contributes to β-cell dysfunction, tissue injury, diabetic complications, and cardio-metabolic disease	[30,41,42,48,55]

## Data Availability

No new data were created or analyzed in this study.
